# Quantifying fetal heart health in gestational diabetes: a new approach with fetal heart quantification technology

**DOI:** 10.3389/fphar.2024.1394885

**Published:** 2024-05-28

**Authors:** Pengjie Zhang, Xinghui Fu, Lijuan Zhao, Lu Wang, Shuning Wu, Yanyan Liu, Jingliang Cheng, Shan Zhang

**Affiliations:** ^1^ Department of Ultrasound, The First Affiliated Hospital of Zhengzhou University, Zhengzhou, Henan, China; ^2^ Henan Vocational College of Nursing, Anyang, Henan, China; ^3^ Department of Ultrasound, Xinyang Traditional Chinese Medicine Hospital, Xinyang, Henan, China; ^4^ Department of Emergency, The Third People’s Hospital of Zhengzhou, Zhengzhou, Henan, China; ^5^ Department of Magnetic Resonance Imaging, The First Affiliated Hospital of Zhengzhou University, Zhengzhou, Henan, China

**Keywords:** gestational diabetes mellitus (GDM), echocardiography, fetal heart quantification, fetal cardiac morphology, cardiac function

## Abstract

**Objective:**

This study aimed to assess the impact of gestational diabetes mellitus (GDM) on fetal heart structure and function using a technique called fetal heart quantification (Fetal HQ), with a focus on mitochondrial dynamics, which employs advanced imaging technology for comprehensive analysis.

**Methods:**

A total of 180 fetuses with normal heart structures, aged 24–40 weeks of gestation, were examined. A 2–3 s cine loop in the standard four-chamber oblique view was captured and analyzed using the speckle-tracking technique with Fetal HQ. Various echocardiographic parameters were evaluated, including four-chamber view (4CV), global spherical index (GSI), global longitudinal strain (GLS), 24-segment spherical index (SI), ventricular fractional area change (FAC), cardiac output (CO), and stroke volume (SV). These parameters were compared between the GDM group and the control group during two gestational periods: 24^+0^ to 28^+0^ weeks and 28^+1^ to 40^+1^ weeks. Statistical analysis was performed using independent samples *t*-tests and Mann-Whitney U tests to identify significant differences.

**Results:**

Twenty fetuses from mothers with GDM and 40 from the control group were recruited at 24^+0^ to 28^+0^ weeks. At 28^+1^ to 40^+1^ weeks, 40 fetuses from mothers with GDM and 80 from the control group were recruited. The fetal left ventricular global longitudinal function was similar between the GDM and control groups. However, compared to the controls, right ventricular function in the GDM group was lower only at 28^+1^ to 40^+1^ weeks. In the GDM group, the global spherical index (GSI) was lower than in the control group at 28^+1^ to 40^+1^ weeks (1.175 vs. 1.22; *p* = 0.001). There were significant decreases in ventricular FAC (38.74% vs. 42.83%; *p* < 0.0001) and 4CV GLS for the right ventricle (−22.27% vs. −26.31%; *p* = 0.005) at 28^+1^ to 40^+1^ weeks.

**Conclusion:**

Our findings suggest that GDM is associated with decreased right ventricular function in the fetal heart, particularly during the later stages of pregnancy (28^+1^ to 40^+1^ weeks), compared to fetuses from healthy pregnancies. The Fetal HQ technique represents a valuable tool for evaluating the structure and function of fetal hearts affected by GDM during the advanced stages of pregnancy.

## Introduction

Hyperglycemia during pregnancy can be classified as either pregestational diabetes or gestational diabetes mellitus (GDM). The American Diabetes Association defines GDM as glucose intolerance first diagnosed during the second or third trimester of pregnancy in women without prior overt diabetes, which resolves postnatally (Association, 2020). GDM is one of the most common pregnancy complications, with an incidence of 11.9% in China, significantly higher than in Japan (6.0%) and South Korea (7.1%) (Nguyen et al., 2018). It is now understood that prolonged exposure of the fetus to elevated glucose levels in the uterus can contribute to congenital developmental abnormalities and structural malformations. Epidemiological studies suggest that offspring of women diagnosed with GDM have an increased risk of developing early-onset cardiovascular disease (CVD) during childhood and young adulthood. These studies also demonstrate that the risk of congenital heart defects in these children increases by more than fivefold (Yu et al., 2019; Smith et al., 2021), potentially due to mechanisms affecting myocardial fiber architecture, cardiac geometry, myocardial deformation, and ventricular function. Additionally, insulin resistance in women with GDM can lead to maternal hyperglycemia and increased glucose transport across the placenta, resulting in fetal hyperinsulinemia, increased metabolic rate, and a tendency for fetal hypoxemia (Paauw et al., 2020).

The fetal heart is one of the major organs affected by hyperinsulinemia and hypoxia (Arslan et al., 2013; Garg et al., 2014). In this regard, animal studies have demonstrated that diabetic intrauterine environments cause fetal myocardial remodeling with dual impacts on the heart (Lehtoranta et al., 2013; Cohen et al., 2017). These impacts include structural heart defects associated with neural crest cells, such as ventricular septal and endocardial defects, Tetralogy of Fallot, and persistent arterial trunks. Additionally, GDM has been associated with hypertrophic cardiomyopathy, pericardial effusion, and bradycardia, even beyond structural malformations. However, it is widely thought that cardiac function impairment during fetal development may precede myocardial thickening (Sonaglioni et al., 2023). Therefore, examining both fetal cardiac morphology and function is crucial in GDM patients. For prenatal evaluation and monitoring of fetal heart function, fetal echocardiography remains the most commonly used method due to its non-invasiveness, affordability, and lack of radiation exposure for both mothers and fetuses. Fetuses of mothers with GDM are in danger of cardiac structural anomalies in the first trimester and cardiac dysfunctions, even cardiac failures, in the second and third trimesters (Lisowski et al., 2010). The fetal heart is affected contributing to perinatal death and stillbirth (Nashaat and Mansour, 2010). Evaluation of ventricular function among fetuses of mothers with GDM is critical in identifying subtle changes to the heart and prompting timely intervention and management.

In addition to traditional Doppler and tissue Doppler ultrasonography, fetal heart quantification (FHQ) is a new ultrasonic technique that enables quantitative analysis of fetal cardiac morphology and ventricular function. This technique uses a dynamic four-chamber view of the fetal heart to provide simultaneous quantification of the size, shape, area, and contractility of both the left and right ventricles. Additionally, FHQ enables segmental analysis of the cardiac ventricles, allowing the detection of changes in function that might go undetected during routine examinations. We hypothesized that fetuses of pregnant women with GDM would exhibit signs of altered cardiac systolic function and shape. We further hypothesized that FHQ could be a simple yet effective tool capable of generating reproducible results in such cases to assess cardiac morphology and function.

This study investigated changes in the end-diastolic global spherical index (GSI) of the four-chamber view (4CV), global longitudinal strain (GLS), 24-segment spherical index (SI), fractional area change (FAC), cardiac output (CO), and stroke volume (SV) in fetuses with GDM. We compared these parameters in GDM fetuses and controls at two gestational periods: 24^+0^ to 28^+0^ weeks and 28^+1^ to 40^+1^ weeks, aiming to understand the effects of GDM on fetal heart function.

## Materials and methods

### Study population

A total of 180 fetuses with structurally normal hearts, aged 24–40 weeks gestational age, were recruited from the ultrasound department of the First Affiliated Hospital of Zhengzhou University in China between May 2023 and January 2024. The participants included 60 fetuses from mothers diagnosed with diabetes and 120 from mothers without diabetes. Inclusive criteria: glucose intolerance first diagnosed during the second or third trimester of pregnancy in women without prior overt diabetes. The OGTT (Oral glucose tolerance test) was performed in 24–28 weeks’ pregnant women recommended by the Chinese Society of Perinatal Medicine as a diagnostic method for GDM, the fasting, 1-h and 2-h postprandial blood glucose thresholds (5.1 mmol/L, 10.0 mmol/L and 8.5 mmol/L, respectively) were measured, and GDM was diagnosed at any time point above the above thresholds. Exclusion criteria were: 1) maternal comorbidities, such as hypertension, renal, or autoimmune diseases; 2) structural or chromosomal fetal anomalies; and 3) fetal growth restriction. Informed consent was obtained from all participants. The study was approved by the research ethics committee of the First Affiliated Hospital of Zhengzhou University (approval number 2019-KY-231), and all patients provided informed consent.

GDM was diagnosed at 24 weeks of gestation using a one-step oral glucose tolerance test (OGTT). Diagnosis required meeting at least one of the following criteria: fasting plasma glucose ≥5.1 mmol/L, 1-h level ≥10.0 mmol/L, or 2-h level ≥8.5 mmol/L. Treatment began with diet and lifestyle modifications, followed by insulin if needed.

### Fetal echocardiography

**FIGURE 1:**
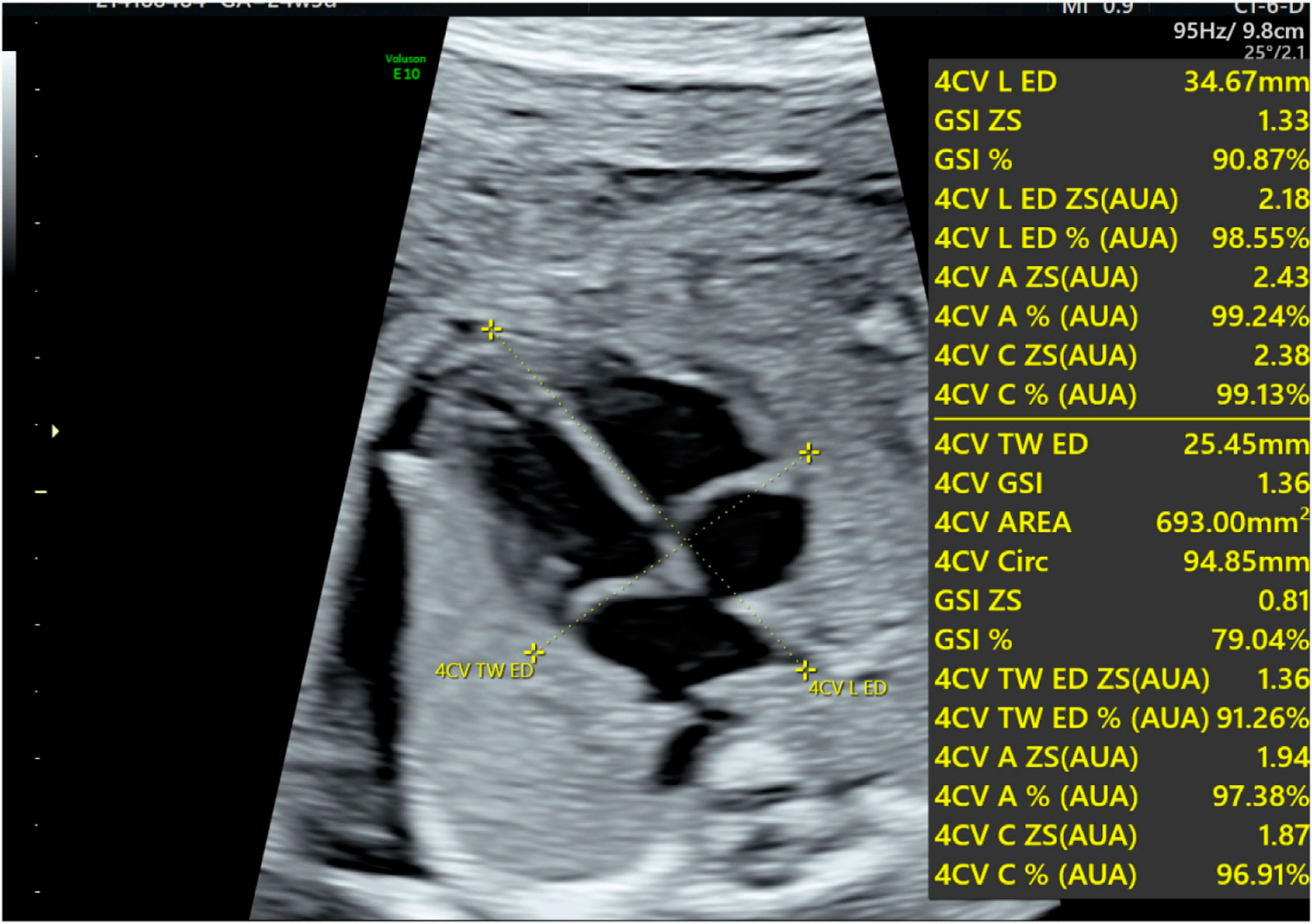
Schematic diagram for the evaluation of fetal four-chamber morphology. The diagram shows the maximum longitudinal transverse diameter of the heart, the global spherical index (GSI), and the area of the four-chamber view.

**FIGURE 2:**
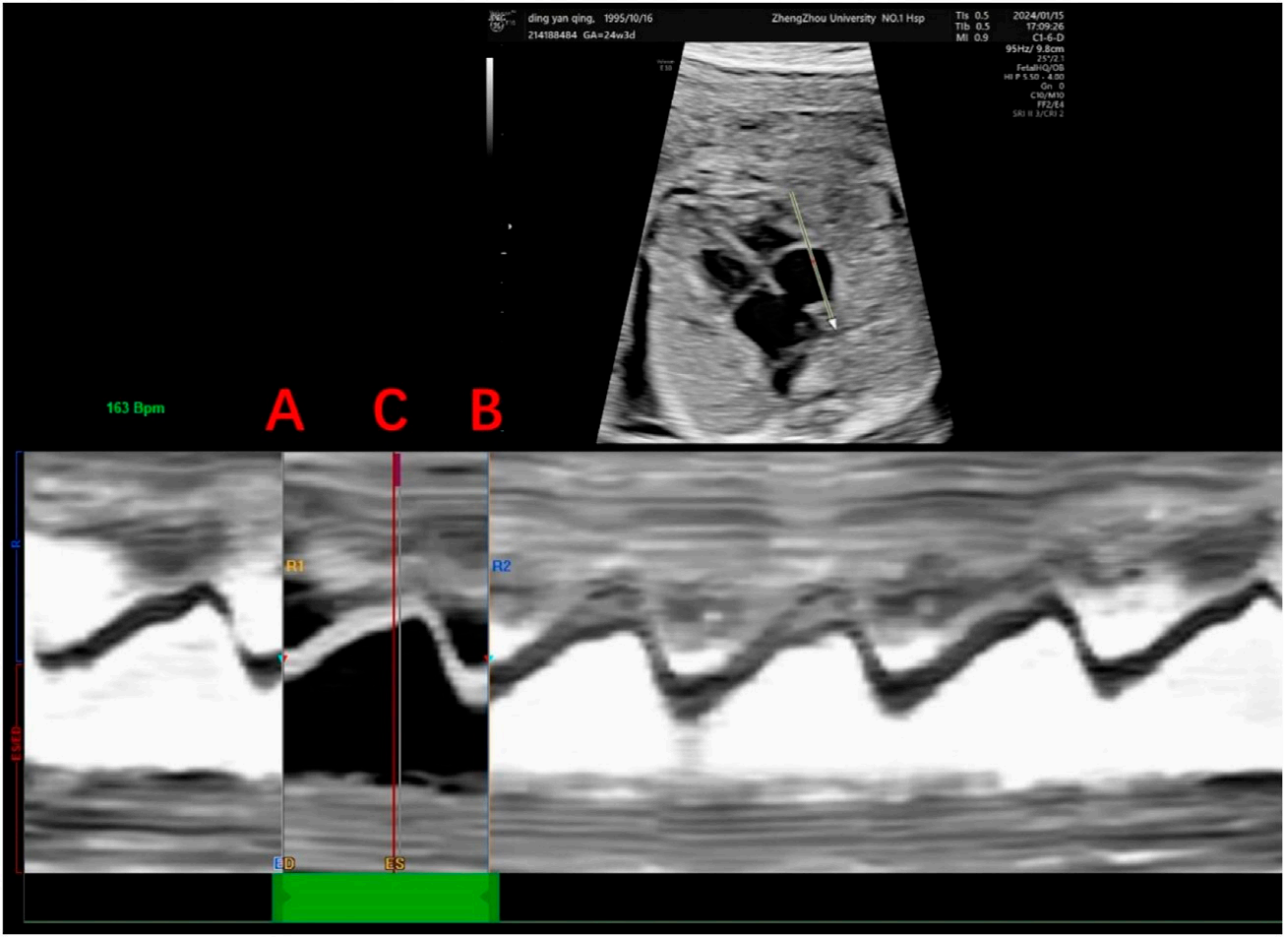
Determination of the cardiac cycle. This figure shows the determination of the right ventricular cardiac cycle, including the end-diastole and end-systole. Anatomical M-mode was drawn in the direction of the apical segment to the basal segment with the sampling line placed at the tricuspid valve to determine a single cardiac cycle, where points A and B represent end-diastole, the first frame of atrioventricular valve closure, and point C represents end-systole, the first frame of atrioventricular valve opening.

**FIGURE 3:**
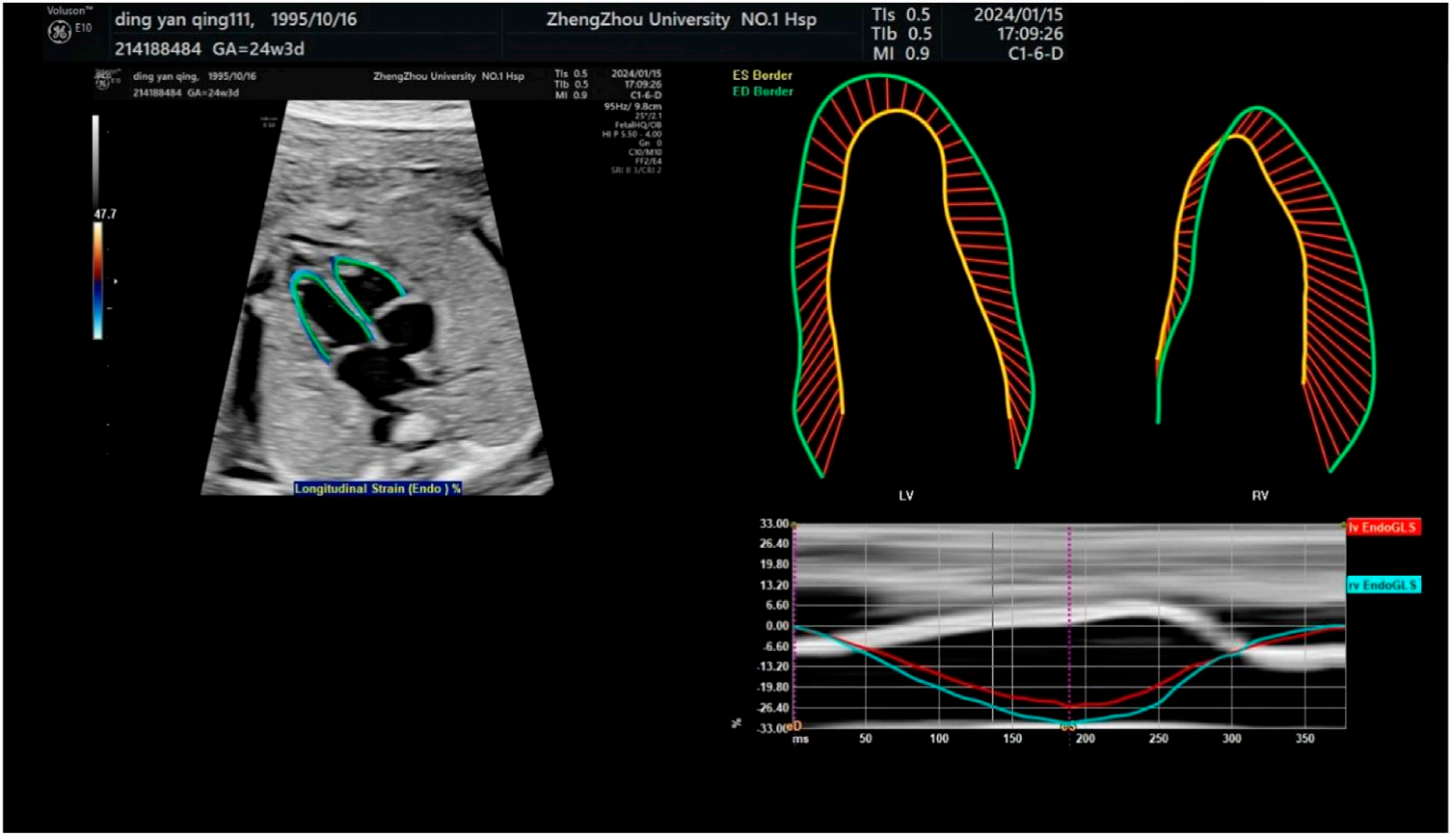
Endocardial tracing of the left and right ventricles and calculation of global longitudinal strain. The upper left image shows a four-chamber view projected obliquely for optimal tracking of endocardial borders. Strain measurements of the left and right ventricles are shown in the upper right.

Echocardiography was performed on all fetuses using a GE Voluson E10 ultrasound system with a 2D/3D volume probe (4–8 MHz). A professional and experienced sonographer captured 3-s dynamic images of the fetal four-chamber view at a frame rate exceeding 80 Hz. The dynamic video was saved in the software system. Image acquisition criteria included: 1) optimal cardiac apex position (3 or nine o’clock), 2) clear visualization of the endocardium, and 3) minimal motion artifacts and rib acoustic shadows (achieved by minimizing fetal movement and maternal breath-holding if necessary). The ultrasound examination focused on morphological and functional heart parameters. First, the maximum longitudinal and transverse diameters of the heart at end-diastole were measured on the four-chamber view from epicardium to epicardium. These measurements were used by the software to automatically calculate the global spherical index (4CV-GSI) and four-chamber view area (Figure 1). Second, end-systolic and end-diastolic images of the cardiac cycle were captured (Figure 2). The endocardial borders on these frames were tracked using speckle tracking technology to obtain morphological and functional parameters of the heart (Figure 3) (DeVore et al., 2016). If necessary, manual adjustments were made to accurately identify the ventricular apices. Finally, data on 4CV global spherical index, global longitudinal strain, 24-segment spherical index, fractional area change, cardiac output, and stroke volume of both ventricles were collected using the software interface (Figure 3).

### Intra- and interobserver variability

A random sample of 20 patients from the study population was measured after 2 weeks by the same examiner and separately by two different examiners. The two examiners who reviewed the US images work at the same institution, and they have been taught a uniform approach to the sonographic description of Fetal HQ. We believe that this may have had a positive effect on the results of our study. Bland-Altman plots were generated to assess interobserver and intraobserver variability. ICC values greater than 0.75 indicated good reliability, and values greater than 0.8 indicated excellent reliability.

### Statistical analysis

Data analysis was performed using SPSS version 26. Continuous variables were assessed for normality and expressed as mean ± standard deviation (SD) if normally distributed or as median (interquartile range) if non-normally distributed. Nominal variables were summarized as frequencies and percentages. Histograms and quantile-quantile plots were used to visually assess the distribution of continuous variables. Comparisons of maternal-fetal characteristics and fetal cardiac parameters between the GDM pregnancy group and the control group were conducted using independent-sample t-tests for continuous variables with normal distributions, Mann-Whitney U tests for continuous variables with non-normal distributions, and chi-square tests for categorical variables.

## Results

### Comparison of clinical data

To study the temporal evolution of fetal cardiac changes in the GDM group, the control and GDM groups were further stratified into second-trimester (24^+0^ to 28^+0^ weeks) and third-trimester (28^+1^ to 40^+1^ weeks) subgroups. In the second trimester, there were 40 patients in the control group and 20 in the GDM group (total 60). No significant difference in gestational age was observed between the groups (25^+0^ vs. 26^+0^ weeks, *p* = 0.11). In the third trimester, there were 40 patients in the GDM group and 80 in the control group (total 120). Again, there was no significant difference in gestational age between the groups (29^+5^ vs. 32 weeks, *p* = 0.568).

### The comparison of fetal cardiac morphology between normal and GDM pregnant women in the second- and third-trimester

**FIGURE 4:**
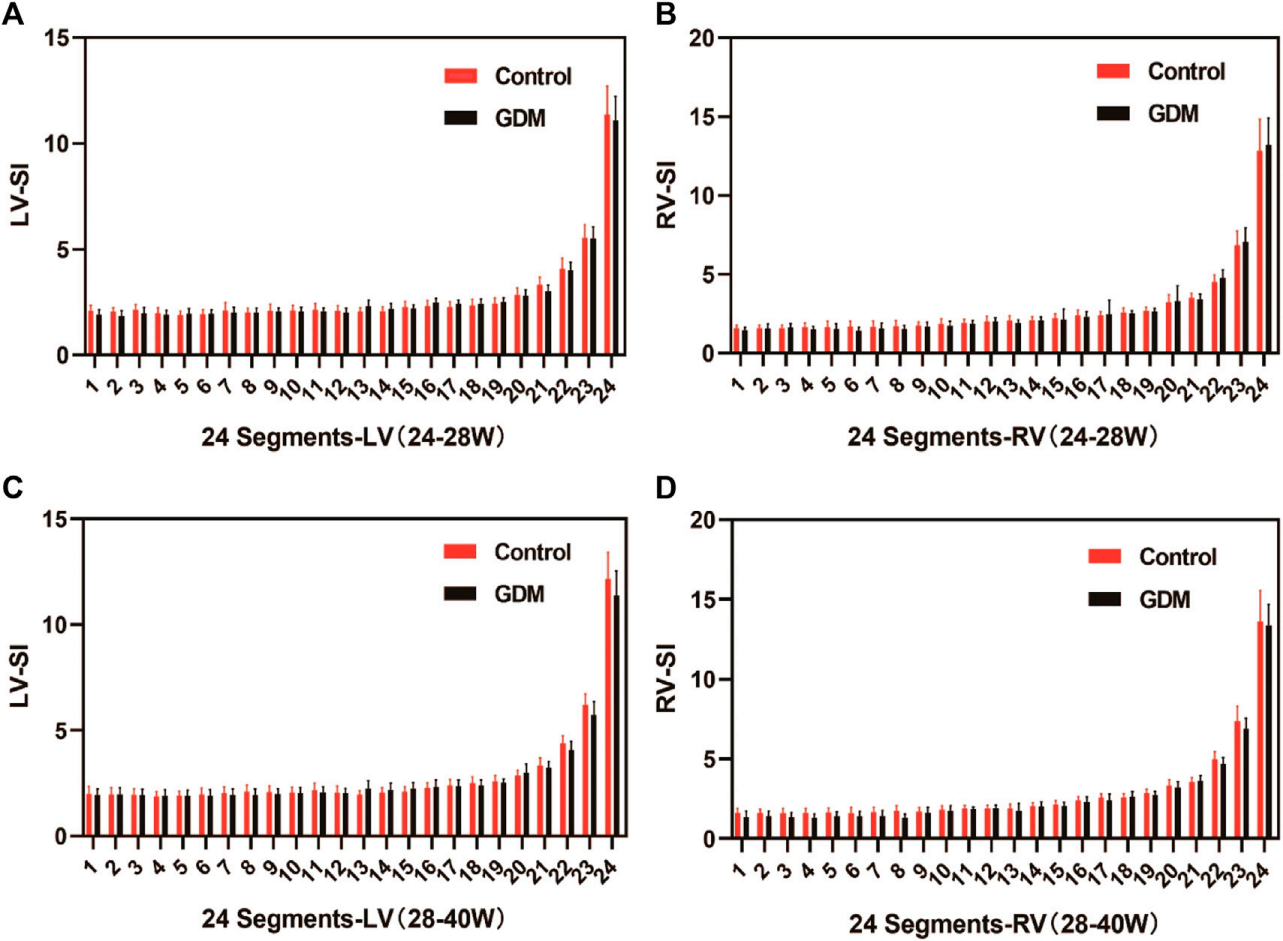
Comparison of the ventricular 24-segment spherical index (SI) between the control and GDM groups at 24+0–40+1 week.**(A)** Left ventricular 24-segment SI values in the GDM and control groups at 24–28^+0^ week (*p* > 0.05). **(B)** Right ventricular (RV) 24-segment SI values in the GDM and control groups at 24–28^+0^ week (*p* > 0.05). **(C)** Left ventricular 24-segment SI values in the GDM and control groups at 28–40^+1^ weeks (*p* > 0.05). **(D)** Right ventricular (RV) 24-segment SI values in the GDM and control groups at 28–40^+0^ weeks (*p* < 0.05).

The fetal 4CV-GSI analysis revealed a significant decrease in GSI in the GDM group compared to the control group at 28^+1^–40^+1^ weeks (*p* < 0.001, Table 1). However, no significant differences were observed in GSI between the two groups during the earlier gestational period (24^+0^–28^+0^ weeks, Table 1, *p* > 0.05). Furthermore, no significant changes were found in 4CV-Area, fetal LV-SI, or RV-SI across the 24 segments throughout pregnancy between the GDM and control groups (Table 1, *p* > 0.05; Figure 4). It is well-established that a decrease in GLS indicates impaired ventricular longitudinal systolic function. Therefore, the findings of our study indicate a reduction in ventricular systolic function in the GDM group compared to the control group, particularly during late pregnancy.

**TABLE 1:** Cardiac morphological paraments of the GDM and control groups.

GDM vs. controls						
	24–28 weeks			28–40 weeks		
Variable	GDM	Controls	*p*	GDM	Controls	*p*
4CV Area (mm^2^)	603.52 ± 44.12	641.61 ± 62.23	0.032	1,078.48 ± 118.05	1,060.81 ± 106.66	0.622
4CV GSI	1.25 ± 0.09	1.29 ± 0.06	0.152	1.13 ± 0.10	1.25 ± 0.08	<0.001*
LV Overall SI	2.21 (12.96–1.46)	2.19 (13.70–1.41)	0.782	2.19 (13.35–1.35)	2.18 (13.99–1.36)	0.579
RV Overall SI	1.96 (15.9–0.83)	2.07 (15.94–0.99)	0.877	1.91 (15.91–0.83)	2.03 (16.23–1.04)	0.333

### The comparison of fetal cardiac function between normal and GDM pregnant women in the second- and third-trimester

**FIGURE 5:**
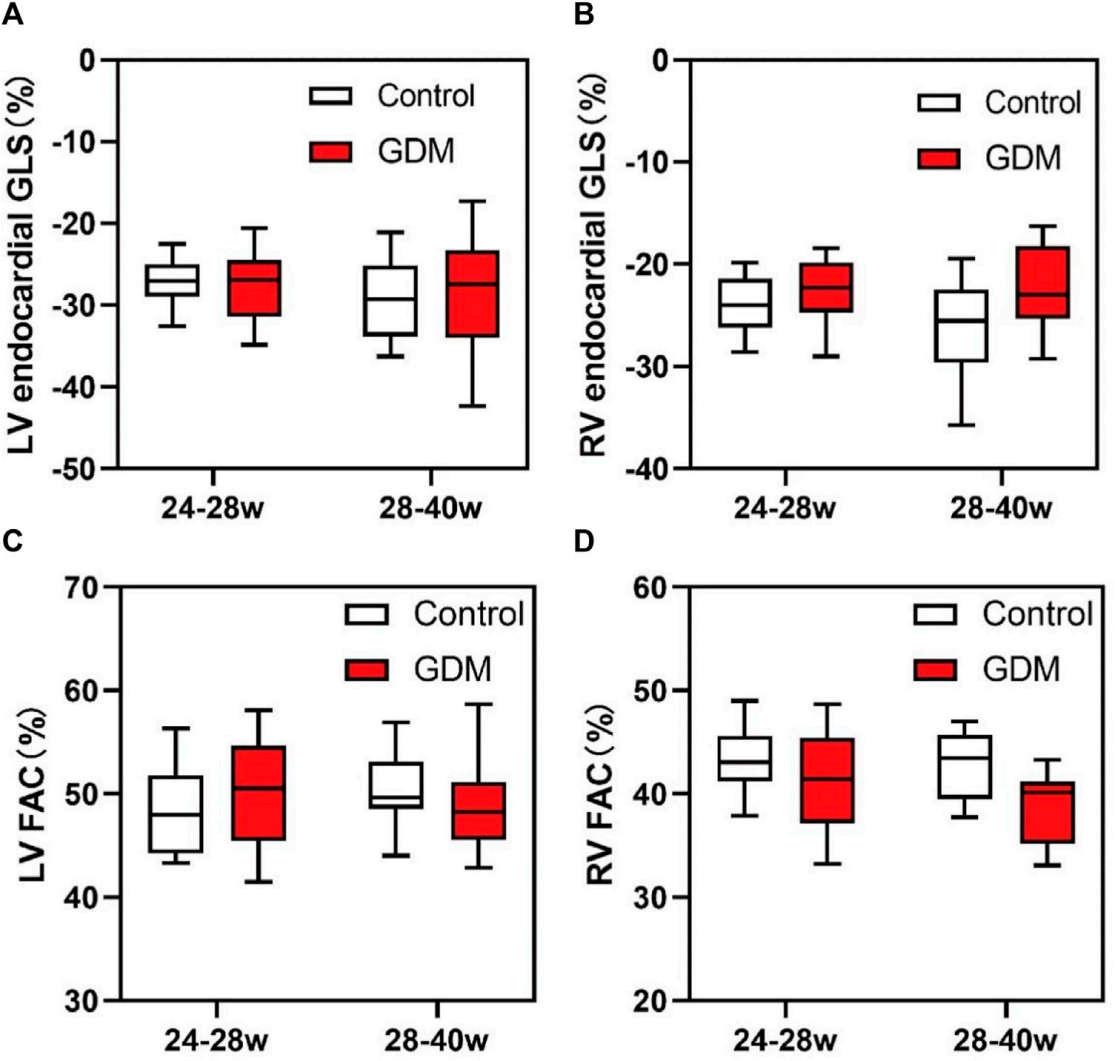
Box plots of global longitudinal strain (GLS) and fractional area change (FAC) in the fetal endocardium. **(A)** Changes in the LV-GLS in the control and GDM groups at 24^+0^ to 28^+0^ and 28^+1^ to 40^+1^ weeks. **(B)** Changes in RV-GLS in the control and GDM groups at 24^+0^ to 28^+0^ and 28^+1^ to 40^+1^ week. **(C)** Changes in the LV-FAC in the control and GDM groups at 24^+0^ to 28^+0^ and 28^+1^ to 40^+1^ week. **(D)** Changes in RV-FAC in the control and GDM groups at 24^+0^ to 28^+0^ and 28^+1^ to 40^+1^ week. Boxes indicate medians and interquartile ranges; whiskers indicate ranges.

No significant differences were observed in fetal LV-FAC, LV-GLS, RV-FAC, RV-GLS, LV-CO, SV, or EF between the second-trimester GDM group and the control group (Table 2; Figures 5A–D, *p* > 0.05).

**TABLE 2:** Cardiac function parameters of the GDM and control groups.

GDM vs. Controls						
	24–28 weeks			28–40 weeks		
Variable	GDM	Controls	*p*	GDM	Controls	*p*
LV-CO/Kg (mL/min)	96.56 (110.50–63.16)	92.80 (162.00–68.11)	0.664	174.26 ± 39.14	190.80 ± 35.88	0.172
EF (%)	65.26 ± 4.85	62.78 ± 3.36	0.07	64.72 ± 4.21	64.57 ± 4.58	0.915
SV (mL)	0.53 ± 0.12	0.56 ± 0.14	0.565	1.21 ± 0.27	1.27 ± 0.29	0.458
LV GLS (%)	−27.42 ± 4.05	−27.10 ± 2.64	0.77	−28.29 ± 7.22	−29.35 ± 4.61	0.62
RV GLS (%)	−22.46 ± 2.85	−24.01 ± 2.72	0.09	−22.27 ± 3.98	−26.31 ± 4.56	0.005*
LV FAC (%)	49.42 ± 5.51	48.57 ± 4.31	0.309	46.75 ± 5.11	48.45 ± 4.60	0.132
RV FAC (%)	41.43 ± 4.80	43.26 ± 3.40	0.116	38.74 ± 3.40	42.83 ± 3.05	<0.001*

However, in the third trimester, fetal RV-GLS and RV-FAC were significantly lower in the GDM group compared to the control group (Figures 5B, D, *p* < 0.05), while no significant changes were observed in fetal LV-GLS or LV-FAC (Figures 5A, C, *p* > 0.05). Additionally, LV-CO, SV, and EF remained unchanged in the third-trimester GDM group (Table 2, *p* > 0.05). These findings suggest that the right ventricle exhibits more pronounced changes compared to the left ventricle, particularly in terms of GLS and FAC parameters. These findings collectively suggest that diabetes predominantly affects fetal heart function in the third trimester, with minimal impact during the second trimester.

### Reproducibility

**FIGURE 6:**
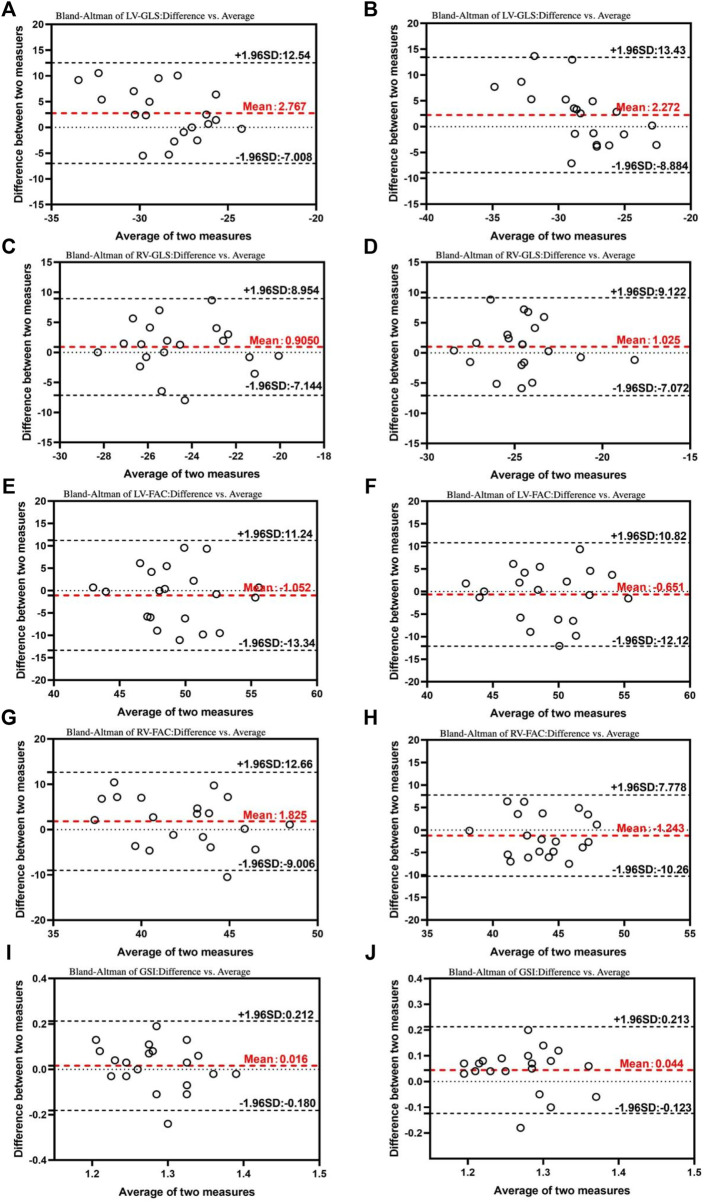
Bland–Altman plots for interobserver and intraobserver variability. The Bland–Altman analysis of the inter- and intraobserver variability of the LV- GLS **(A)**, RV-GLS **(C)**, LV-FAC **(E)**, RV-FAC **(G)**, and GSI **(I)** in 24^+0^-28^+0^ weeks. The Bland–Altman analysis of the inter- and intraobserver variability of the LV- GLS **(B)**, RV-GLS **(D)**, LV-FAC **(F)**, RV-FAC **(H)**, and GSI **(J)** in 28^+1^-40^+1^ weeks. This results demonstrated that the mean variability in the inter- and intraobserver results was acceptable. LV, left ventricle; GLS, global longitudinal strain; RV, right ventricle; FAC, fractional area change; GSI, global spherical index.

Both intra-observer and inter-observer comparisons showed excellent reliability (ICCs >0.9) for all parameters, as evidenced by Table 3. Bland-Altman plots (Figure 6) further demonstrated good agreement between repeated measurements by the same observer and between measurements by different observers. These results indicated that the Fetal HQ technique offers high repeatability and ease of use. Skilled ultrasound professionals can typically complete a fetal heart analysis within 5 min. Moreover, the software’s semi-automated endocardial analysis procedure ensures good intra- and inter-observer reproducibility. In conclusion, the Fetal HQ technique provides an accurate and efficient means for evaluating fetal heart function, independent of the four-chamber view’s apical orientation. This represents a valuable new and simple diagnostic tool for fetal heart assessment.

**TABLE 3:** Intra-observer and inter-observer correlation.

Intra-observer			inter-observer	
Measurements	ICC	95% confidence interval	ICC	95% confidence interval
LV-GLS	0.956	0.893–0.987	0.958	0.823–0.986
RV-GLS	0.964	0.911–0.985	0.965	0.914–0.986
LV-FAC	0.962	0.907–0.985	0.908	0.721–0.947
RV-FAC	0.955	0.885–0.983	0.919	0.825–0.988
4CV-GSI	0.925	0.863–0.994	0.936	0.865–0.995

ICC, intra-class correlation coefficients; GSI, global spherical index of LV, left ventricle and RV, right ventricle; FAC fractional area change of LV, left ventricle and RV, right ventricle, 4CV, four-chamber view.

## Discussion

Gestational diabetes mellitus, defined as the development or first onset of glucose intolerance during pregnancy, is a prevalent endocrine metabolic disorder affecting pregnant women (Choudhury and Rajeswari, 2021). Current evidence suggests that an intrauterine hyperglycemic environment can directly cause fetal cardiomyocyte enlargement and disrupt myofibrillar arrangements, leading to fetal cardiac enlargement and myocardial remodeling (Cerychova and Pavlinkova, 2018). Animal studies have shown that adverse cardiac remodeling changes, including dysregulation of insulin-like growth factors 1 and 2 (IGF-1 and IGF-2), increased collagen synthesis, fibrosis, and apoptosis, contribute to cardiac hypertrophy, ventricular dilatation, and myocardial dysfunction in GDM fetuses (Lin et al., 2017). Therefore, deterioration of clinical markers of cardiac function often serves as the first sign of fetal pathology. Prenatal assessment of fetal ventricular function using ultrasound is crucial for identifying subtle cardiac changes and enabling timely intervention and management.

Utilizing the advanced Fetal HQ technique, this study delved into the morphology and function of fetal ventricles in GDM mothers. Key measurements like ejection fraction (EF), left cardiac output, and GLS were taken. Notably, biventricular function in GDM mothers mirrored controls at 24^+0^ to 28^+0^ weeks. However, fetuses of GDM mothers displayed a decrease in GSI at 28^+1^ to 40^+1^ weeks, suggesting a more spherical heart shape. Furthermore, this later period witnessed a decline in right ventricular systolic function, evidenced by reduced RV-GLS, RV-FAC, and RV-SI across all segments. Studies in human and lamb models suggest that the right ventricle plays a dominant role in fetal development, contributing approximately two-thirds of total cardiac output (Rudolph and Heymann, 1970; Meah et al., 2016). The majority (75%–90%) of this output is shunted to the systemic circulation through the arterial duct (Mielke and Benda, 2001). This emphasizes the critical importance of right ventricular morphology and function in fetal life support. Since the fetal heart depends on glucose metabolism, hyperglycemia may cause the fetus’s metabolism to accelerate, and induced hypoxia and increased oxidative stress would initially harm the RV, which may account for decreased cardiac performance (Gireadă et al., 2022). Several studies have investigated the impact of GDM on fetal heart function. Song et al. (2021) found that fetuses of GDM mothers exhibited altered cardiac morphology, including reduced GSI (indicating a more spherical heart shape) and impaired right ventricular systolic function (evidenced by decreased FAC and total strain) in both the second and third trimesters. Yovera et al. (2021) reported: 1. Reduced right ventricular function in fetuses of GDM mothers at both 24^+0^ to 32^+0^ and 32^+1^ to 40^+1^ weeks, observed through decreased function in all right ventricle segments; 2. Lower GSI only at 32^+1^ to 40^+1^ weeks. 3. No overall change in left ventricular systolic function indices, but reduced longitudinal function and decreased basal segment contractility identified through segmental analysis. Miranda et al. (2018) studied 76 fetuses of GDM mothers at 30–33^+6^ weeks using conventional TDI and STI echocardiography. They found evidence of biventricular diastolic insufficiency and right ventricular systolic insufficiency in third-trimester GDM fetuses. These findings are inconsistent with our results. This inconsistency could stem from variations in subject selection, maternal factors, diabetes control, image acquisition protocols, and software employed for fetal cardiac function analysis.

GLS and FAC reflect cardiac systolic function. Longitudinal strain indicates myocardial deformation from the base to the apex. During systole, ventricular myocardial fibers shorten as they move from the base to the apex, and longitudinal strain is the first to show exceptions and abnormal strain rates in fetal cardiac dysfunction; As the fetal heart requires glucose for energy, when ischemia or hypoxia occurs, the endocardium is the first to be injured coupled with a decrease in GLS, making it a sensitive indicator of cardiac dysfunction in the early stages (Lee and zhang, 2021). A low RV-GLS indicates impaired longitudinal systolic function in the right ventricle. Due to its unique triangular shape in the coronal plane and crescent shape in the transverse plane, calculating the right ventricle’s ejection fraction using the Simpson method isn’t feasible. As an alternative, measuring the FAC from a four-chamber view provides valuable insights into RV function (DeVore et al., 2019). A decrease in RV-FAC signifies a decline in overall right ventricular systolic function. Importantly, our study found no abnormalities in LV systolic function (LV-GLS and LV-FAC) at either gestational age (24^+0^–28^+0^ or 28^+1^–40^+1^ weeks), as evidenced by the absence of significant differences between the GDM and control groups. Interestingly, RV systolic dysfunction emerged only at 28^+1^–40^+1^ weeks. This might be attributed to the dominant role of the right ventricle in the fetal cardiovascular system. It has been established that that hyperglycemia during pregnancy negatively impacts fetal right ventricular function, but the effect on the left ventricle remains unclear due to conflicting findings across studies.

We opted for Fetal HQ technology for its detailed fetal cardiac evaluation capabilities. This technology leverages speckle tracking technology, offering not only a comprehensive assessment of fetal heart size and shape but also a 24-segment analysis of both ventricles, enabling precise and quantitative evaluation of their systolic function. Previous studies (Comas et al., 2010; Popescu et al., 2022) had shown that subtle fetal cardiac changes in GDM patients often go undetected by conventional Doppler techniques. Fetal HQ technology, a non-invasive quantitative ultrasound method, overcomes the limitations of the four-chamber view’s apical orientation and accurately quantifies comprehensive fetal cardiac function through specific values of various independent biological parameters (e.g., GSI, GLS, FAC). This provides a new diagnostic approach for determining fetal heart function and identifying subclinical segmental alterations beyond structural malformations (Mondillo et al., 2011; Luis et al., 2019; Lisi et al., 2022). In our study, compared to the control group, fetuses in the GDM group exhibited no significant changes in CO, EF, or SV during either the second or third trimester. However, reduced RV-GLS, RV-FAC, and GSI were observed in the GDM group only in the third trimester. This may be related to the increasing nutritional demands and workload on the right ventricle as gestational weeks increase (Lei et al., 2018). The semi-automated nature of this technology ensures good intra- and interobserver reproducibility, consistent with our findings (Dickson et al., 2017). However, given the small size and fast heart rate (120–160 bpm) of the fetal heart, speckle-tracking imaging is crucial to minimize variability in the results by achieving accurate tracking with a high frame rate (at least 80 Hz). Next, we evaluated two independent gestational windows to explore the relationship between cardiac morphology and function in normal and GDM fetuses across the second and third trimesters. No abnormalities were found in second-trimester fetuses of the GDM group, while subclinical changes in morphology and function emerged only in third-trimester fetuses.

Recent studies have highlighted the role of mitochondrial dysfunction in the pathogenesis of cardiac abnormalities in fetuses of GDM mothers. Mitochondria are crucial for energy production and metabolic regulation in cardiomyocytes, and their dysfunction can lead to impaired cardiac function and structural changes. In GDM, hyperglycemia-induced oxidative stress can damage mitochondrial DNA and disrupt the normal function of mitochondrial respiratory chain complexes, leading to decreased ATP production and increased reactive oxygen species (ROS) formation. This mitochondrial stress can trigger cardiomyocyte apoptosis and contribute to the observed cardiac remodeling and dysfunction in GDM fetuses. Furthermore, mitochondrial dynamics, including fission and fusion processes, are essential for maintaining mitochondrial and cardiac health. Imbalances in these dynamics, often observed in diabetic conditions, can result in fragmented and dysfunctional mitochondria, further exacerbating cardiac dysfunction. Therefore, the assessment of mitochondrial parameters, such as mitochondrial DNA copy number, oxidative stress markers, and expression levels of proteins involved in mitochondrial dynamics, could provide additional insights into the mechanisms underlying cardiac alterations in GDM fetuses.

This study leveraged the strengths of the Fetal HQ technique, which offers high image quality, high frame rates, and the ability to detect subtle changes in the fetal heart. Additionally, Fetal HQ-derived indicators are more accurate than traditional ones and free from directional interference. Despite strict selection criteria, potential selection bias within the study population cannot be entirely ruled out. A larger cohort of fetuses is warranted to definitively assess the effectiveness of GSI, FAC, and GSL in detecting fetal cardiac function in GDM mothers. While no significant abnormalities were detected in the 24-segment SI of either ventricle, third-trimester fetuses of GDM mothers exhibited impaired right ventricular systolic function, evident in low FAC and GLS values. A low GSI further suggests overall fetal heart dilatation.

This suggests its potential for predicting future cardiovascular complications. The effect of gestational diabetes mellitus on fetal growth and development is mainly manifested as affecting fetal heart development, affecting fetal peripheral blood flow circulation at the same time, resulting in increased peripheral vascular resistance. The increase of blood flow resistance of organs that rely on peripheral blood vessels to provide blood perfusion, such as kidney, is easy to lead to fetal congenital heart failure, renal insufficiency, etc. Severe disease can lead to intrauterine death of the fetus. Therefore, early evaluation of the development of fetal heart, kidney and other important organs and formulating corresponding treatment measures are of great significance to improve the prognosis of pregnant women and fetuses. However, several limitations warrant consideration. Firstly, the study lacked case groups with poor glycemic control, potentially limiting the generalizability of findings. Secondly, the smaller sample size in the second trimester compared to the third trimester raises concerns about the conclusiveness of results for that period. Finally, the lack of validation of cardiac function in newborns after birth leaves unanswered whether the observed changes in the GDM group persist beyond pregnancy.

The present study employed the Fetal HQ technique to investigate the impact of diabetes mellitus on fetal cardiac function. Notably, right ventricular function parameters (GLS and FAC) were reduced, and overall heart morphology became more rounded at 28^+1^–40^+1^ weeks. This difference aligns with the increased importance of right ventricular function in the third trimester. Future studies will address the limitations of this research to enhance comprehensiveness and robustness.

## Conclusion

Our study demonstrated an association between GDM and reduced fetal right ventricular function, primarily observed between 28^+1^ and 40^+1^ weeks of gestation. Furthermore, the Fetal HQ technique proved to be a feasible and effective approach for assessing both morphology and function in the hearts of GDM fetuses during late pregnancy.

## Data Availability

The raw data supporting the conclusion of this article will be made available by the authors, without undue reservation.
