# Supplementary material for: Quantifying fetal heart health in gestational diabetes: a new approach with fetal heart quantification technology
Source: Front Pharmacol. 2024 May 28;15:1394885. doi: 10.3389/fphar.2024.1394885 (PMC11165031; doi:10.3389/fphar.2024.1394885)
Supplement: Supplementary file 1 [file DataSheet2.PDF]

Informed Consent Form for Fetal Echocardiography

Dear Expectant Mother,

Thank you for your trust! Please carefully read the following information before undergoing prenatal ultrasound examination, so that you have an objective understanding of the limitations of prenatal ultrasound examination. You have the right to withdraw from prenatal ultrasound examination before the examination is carried out!

Fetal echocardiography is a specialized examination for the fetal heart, aimed at screening for serious heart malformations through ultrasound examination.

There are certain limitations to fetal echocardiography:
(1) The size and hemodynamics of the fetal heart and related vessels change as the fetus develops. The results of each ultrasound examination only represent the growth and development level at that time.
(2) Fetal malformation development is a dynamic process. Without reaching a certain stage of development, malformations may not be detected through ultrasound examination.
(3) Some diseases may not show abnormalities in mid-pregnancy ,but may appear in late pregnancy, such as small ventricular septal defects, valve abnormalities, and pulmonary artery stenosis.
(4) Significant changes in cardiac hemodynamics occur after birth, and prenatal diagnosis may not correspond with postnatal diagnosis, such as premature closure of the oval foramen, early closure of the ductus arteriosus, or aortic narrowing. The condition may either worsen or improve.
(5) Due to factors such as instrument conditions, gestational age, fetal position, amniotic fluid, fetal movement, bone shadows, and maternal obesity, some structures may not be displayed or may be displayed unclearly.
(6) The following developmental abnormalities may not be detected or cannot be definitively diagnosed, including small ventricular septal defects, moderate valve and vascular stenosis, various valve diseases without significant regurgitation, some types of atrial septal defects, pulmonary vein drainage anomalies, aortic arch malformations, coronary artery diseases, aorticopulmonary window, myocardial diseases, and cardiac tumors, etc.

The ultrasound examination methods currently recommended follow internationally recognized safety standards. The statement "no obvious abnormalities observed" in

the ultrasound examination result does not mean "everything is normal". The content of "ultrasound description" in the main examination report of this ultrasound examination does not cover fetal structures not described.

Under current conditions, the detection rate and diagnostic compliance of any expert or instrument cannot reach one hundred percent. When the examination effect cannot be achieved due to fetal position, repeated examinations may be necessary. The examination results may be limited if the gestational age is inappropriate. We hope that the expectant mother and family can understand this.

The doctor has fulfilled the obligation to inform, and I have understood and agreed to undergo the above examination and assume the corresponding risks. To confirm that the above content represents the true intention of both parties, I hereby sign below.

Please write "I have understood and agreed to the above content" in the box below.

[Signature of Physician]
[Signature of Patient]
Contact Information:
Date:
