# Supplementary material for: Quantifying fetal heart health in gestational diabetes: a new approach with fetal heart quantification technology
Source: Front Pharmacol. 2024 May 28;15:1394885. doi: 10.3389/fphar.2024.1394885 (PMC11165031; doi:10.3389/fphar.2024.1394885)
Supplement: Supplementary file 2 [file DataSheet1.PDF]

胎儿超声心动图知情同意书

尊敬的孕妇朋友：

感谢您的信任！请您在进行产前超声检查前，仔细阅读以下告知，以便对产前超声检查的局限性有客观的认识。**您在检查实施前，有退出产前超声检查的权利！**

一、胎儿超声心动图是针对胎儿心脏的专项检查，旨在通过超声检查筛查出严重的心脏畸形。

二、胎儿超声心动图检查存在一定的局限性。（1）胎儿心脏以及相关血管的径线及血流动力学会随着胎儿发育而变化，每次的超声检查结果只代表当时的生长发育水平。（2）胎儿畸形发展是一个动态的过程，在没有发展到一定程度时，超声检查是不能发现的。（3）部分疾病在中孕期未显示异常，但在晚孕期可能出现异常，如小的室间隔缺损、瓣膜的异常、肺动脉狭窄等。（4）产后心脏血流动力学会发生巨大改变，产前诊断可能与产后诊断不符合，如卵圆孔早闭、动脉导管早闭、主动脉缩窄，病情可能变轻或加重。（5）受仪器条件或其他各种因素影响，包括孕周、胎儿体位、羊水、胎动、骨声影、孕妇肥胖等，一些结构可能无法显示或显示不清。（6）以下发育畸形可能无法发现或不能明确诊断，包括较小的室间隔缺损、中度的瓣膜及血管狭窄、各种无大量返流的瓣膜疾病、部分型房间隔缺损、肺静脉异位引流、主动脉弓畸形、冠状动脉疾病、主肺动脉窗、心肌疾病及心脏肿瘤等。

五、目前推荐采用的超声检查方法均遵照国际公认的安全性标准进行。本次超声检查结果"未见明显异常，"不代表"一切正常"，本次超声检查主要检查报告中"超声描述"的内容，没有描述的胎儿结构不在本次超声检查范围内。

七、就目前条件来说，任何专家、任何仪器的检出率与诊断符合率均不能达到百分之百，当由于胎儿体位关系而达不到检查效果时，有可能进行反复检查，如孕周不适，检查结果可能受限。望孕妇及家属理解。

医生已经履行告知义务，我对上述内容已经知晓并理解，同意接受上述检查并承担相应风险。为确认上述内容为双方意愿的真实表达，现签字生效。

请在下方方框内书写**"我对上述内容已经知晓并理解"** 。

医师签名:

受检者签名:

联系方式:

年　　月　　日
